# TAXODIUM Version 1.0: A Simple Way to Generate Uniform and Fractionally Weighted Three-Item Matrices from Various Kinds of Biological Data

**DOI:** 10.1371/journal.pone.0048813

**Published:** 2012-11-21

**Authors:** Evgeny V. Mavrodiev, Alexander Madorsky

**Affiliations:** 1 Florida Museum of Natural History, University of Florida, Gainesville, Florida, United States of America; 2 Department of Physics, University of Florida, Gainesville, Florida, United States of America; Biodiversity Insitute of Ontario – University of Guelph, Canada

## Abstract

An open-access program for generating three-item statement (3TS) matrices from data such as molecular sequences does not currently exist. The recently developed LisBeth package allows for representation of hypotheses of homology among taxa or areas directly as rooted trees or as hierarchies; however, LisBeth is not a standard matrix-based platform. Here we present “TAXODIUM version 1.0” (TAXODIUM), a program designed for building 3TS-matrices from binary, additive (ordered) and non-additive (unordered) multistate characters, with both uniform and fractional weighting of the statements. TAXODIUM also facilitates, for the first time, use of Maximum Likelihood analyses with 3TS matrices, but future implementation of the 3TS analysis in a statistical framework will require more exploration.

## Introduction

Three-taxon statement (3TS) analysis was originally established as a putatively more precise use of parsimony [1], [2]; it was designed for use with binary characters [2]. 3TS representation reduces information about taxon relationships to a series of three-taxon statements (3TSs) in the form A(BC): taxa B and C are more closely related to each other than to taxon A [2]. The 3TSs, as implemented in data matrices, are high-level hypotheses concerning the relationships of two taxa relative to a third, and are not low-level hypotheses about character state distribution within a standard matrix [3]. Therefore, 3TS data are an entirely different way of viewing information, and represent relationships directly [4]. We believe each character in a conventional data matrix (e.g. binary, ordered multistate, molecular, etc.) can be represented as a series of 3TSs. However, there are no open-access programs currently available to generate 3TS matrices from data such as molecular sequences. The recently developed LisBeth package, for example, allows for explication of hypotheses of homology among taxa or areas directly, either as rooted trees or as hierarchies [5]. However, LisBeth is not a standard matrix-based platform.

## Results

We developed “TAXODIUM version 1.0” (TAXODIUM), a program for generating 3TS-matrices from binary (**b**), additive (ordered) (**omc**) and non-additive (unordered) multistate characters (**umc**) (i.e. up to 25 symbols, representing IUB/IUPAC codes for DNA, RNA, and AAs), and with optional features for uniform or fractional weighting of the resulting statements.

TAXODIUM has a simple command line interface, which is described in Supplement S1. The utility is written in portable C++, and the source code compiles equally well with Microsoft Visual C++ 2010 Express and the GNU C++ compiler version 3.4.4 available from Cygwin. Porting this code to any other platform with a standard C++ compiler should be possible.

**Figure 1:**
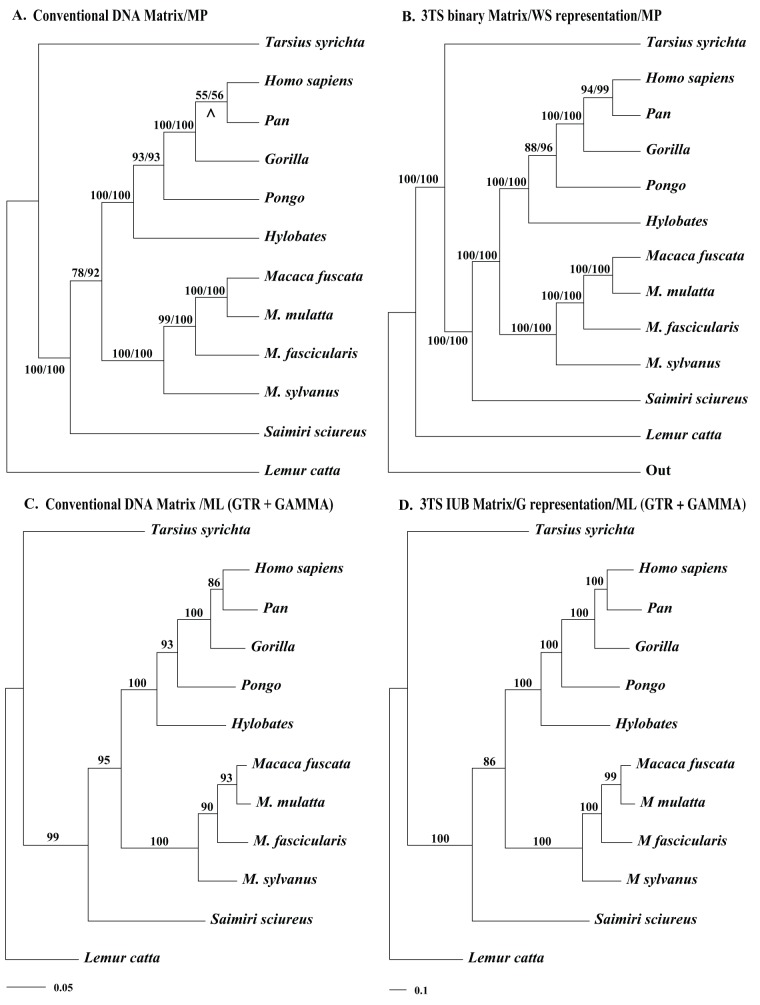
Example Maximum Parsimony (**MP**) **and Maximum Likelihood** (**ML**) **analyses of the conventional and 3TS represented clock-like umc-matrices.** **A.** One of the two most parsimonious trees (length  = 1153, CI  = 0.6496, RI  = 0.5960) recovered from a MP analysis of the clocklike mt *NADH*-dehydrogenase subunit-4 (*NADH*-4) (Hayaska et al., 1988, summarized in [15]). The matrix comprised a total of 898 nucleotide characters, of which 367 were parsimony informative. All characters were treated as “unordered” (Fitch parsimony). The MP analysis was conducted in PAUP* 4.0b10 [15], with an heuristic search with 1000 random addition replicates (saving no more than 100 trees per replicate), and the TBR branch swapping/MulTrees option in effect [15]; gaps were treated as “missing” data. Branches with a minimum length of zero were collapsed. Bootstrap (MP BS)/Jackknife (MP JK) values, respectively, are provided above or below the branches, and indicate nodes with greater than 50% support. Both MP BS/JK estimates were obtained using 100 replicates and 100 random addition sequences (saving no more than 1000 trees per replicate), with the TBR branch swapping/MulTrees option in effect. In total, 36.0% of the characters were deleted in each JK replicate. *Lemour catta* selected as the outgroup. **B.** The single, most parsimonious tree (length  = 26305, CI  = 0.8142, RI  = 0.07718). recovered from an MP analysis of a 3TS binary matrix generated by TAXODIUM from the *NADH*-4 matrix (Figure 1A). See the Figure 1A legend for the details of MP analysis. Williams-Siebert (WS) representation was performed, and the value of the outgroup was fixed as *Lemour catta*; all 3TS were uniformly weighted (command: **taxodium.exe input_file_name.csv –idna –ob –og –nex**). The number of characters (3TSs) was 21418, of which all were parsimony informative. **C.** The Maximum Likelihood (ML) tree (log likelihood  = −5722.341070) inferred from the *NADH*-4 matrix (Figure 1A) with RAxML [16]. A total of 500 rapid bootstrap (ML BS) replicates were used to assess support for individual nodes. GTR+ G substitution model assumed as a best choice. **D.** The ML tree (log likelihood  = −208795.522546) inferred from the IUB-notated 3TS matrix generated by TAXODIUM from the *NADH*-4 matrix (**Fig**ures 1A, C). See the Figure 1C legend for additional details of ML analysis. G-representation was performed; **an operational outgroup was not explicitly defined and, therefore, was not included in the analysis** (command: **taxodium.exe input_file_name.csv –idna –odna –phy**). The total number of characters (3TSs) in the 3TS matrix was 61043.

TAXODIUM accepts input data files in Comma Separated Value (CSV) format. These files can be generated with programs such as Excel, OpenOffice Calc, etc., as well as with Mesquite [6]. Output data files can be written in simplified NEXUS and PHYLIP formats, as well as in CSV format (Supplement S1, Examples S1–S3) and successfully used by standard phylogenetic software (Figure 1).

Currently, the maximum number of taxa or characters in the input matrix must not exceed 5000 or100000, respectively. However, these values can be modified within the source code if necessary (See Supplement S1 for details). TAXODIUM is freely available from http://www.phys.ufl.edu/~madorsky/taxodium.

## Analysis

The MS-DOS program TAX (incl. MATRIX and MOMATRIX) [7], [8], [9] renders a matrix of standard characters into a matrix of three-item statements, creating appropriate output for analysis with parsimony programs such as Henning86 and NONA [8]. Nelson and Ladiges, however, did not address the issue of **umc** [8], and the representation of **umc**-matrices as a series of 3TSs remains a subject of discussion [10], if possible et all [10].

Nelson and Platnick [2] mentioned that it might be necessary to examine separately all possible orderings (and the 3TSs that each implies) in order to extract all possible information from **umc** distributions. Later, Nelson and Ladiges [9], and Williams and Ebach [4] suggested that, from the perspective of 3TS-analysis, a multi-state character is equivalent to a suite of 3TSs in which no statements appear more than once.

**Figure 2:**
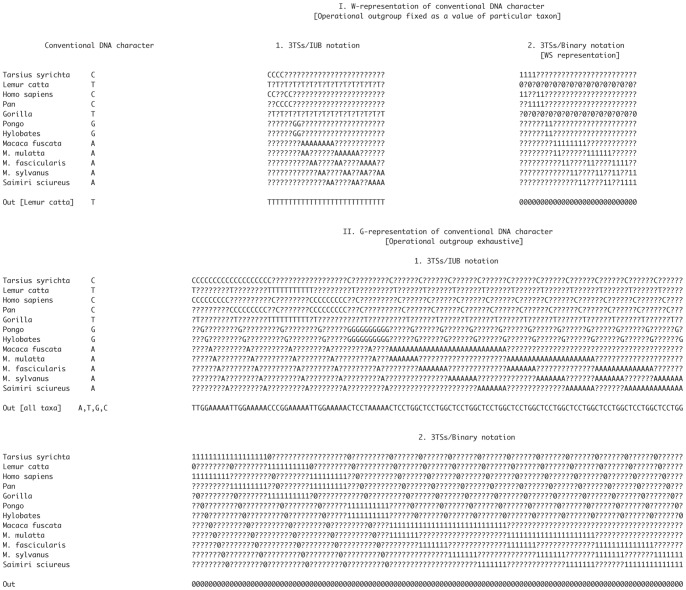
W and G-representations of DNA character as implemented in TAXODIUM. Conventional DNA character from Hayaska et al (1988), summarized in [15], see matrix “primate-mtDNA.nex”, character 773.

In TAXODIUM, we attempted to implement the simplest 3TS representation of **umc** by **exhaustion of the outgroup value** (Figure 2, Example S2). Within the resulting 3TS matrix, each taxon is represented with all possible “minimal” relationships with all other taxa of the same matrix. We call this a “General representation” (**G**).

Williams and Siebert [8] (see also Nelson and Platnick [2]) pointed out that the 3TS approach estimates *a priori* a putative synapomorphy or codes the data relative to outgroups defined *a priori*. Therefore, another way to build a 3TS-matrix is to code standard data relative to a **fixed value of the outgroup** (Figure 2, Example S2). This method of 3TS representation of **umc** is called “Williams'” (**W**). Both **G** and **W** methods do not require the initial non-additive re-coding of the standard **umc** characters [11].

TAXODIUM represents **b** characters as 3TSs as described in [2] (Example S1).

We call a matrix “binary” if it contains states 0/1, and state 0 is assumed to be plesiomorphic *a priori* [2]. Without the latter assumption, a 0/1 matrix is a particular type of **umc**-matrix. Therefore, by using both **G** and **W** methods, we can represent the **umc**-matrix as a 3TS-matrix in two ways:

1. We can maintain multistate notation in the 3TS matrix, or.
2. We can represent the 3TS matrix as a binary matrix.

Both ways are implemented in TAXODIUM (Example S2). When analyzed with standard parsimony analysis, binary or multistate-notated 3TS matrices produce either similar or identical results. However, this may not necessarily be the case with other methods for phylogeny reconstruction (Figures S2, S3).

All kinds of 3TS representations of molecular characters are very sensitive to conventional alignment quality and, therefore, they must be handled with extreme caution to ensure accuracy. The 3TS approach, therefore, may help to test the quality of the standard molecular data.

Fractional weighting (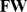) [7], [9] is also implemented in TAXODIUM to compensate for the influence of putatively redundant statements. In the case of **umc**, the fractional weighting of the resulting 3TSs is challenging, and may be a subject that warrants future consideration.

In the case of **G**-representation of **umc**, the following formulas from [9] have been modified accordingly:
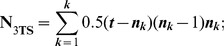


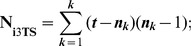


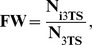
where 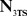 is the total number of 3TSs, 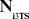
is the number of independent 3TSs, ***t*** is a number of taxa, ***n*** is the number of taxa with the informative states, and ***k*** is the number of informative states of **umc**.

In the case of **W**-representation of **umc**, we included an optional weight (Wt) to the final 3TSs using the following fraction:
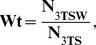
where 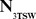 is the total number of 3TSs if the value of the outgroup is fixed. This procedure is different from fractional weighting, as originally proposed by Nelson and Ladiges [9]. We recommend using the 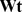 option for the clock-like **umc**-matrices.

There are issues in the fractional weighting of 3TSs from **omc** (e.g., [12], [13], [14]). The current version of TAXODIUM can represent the additively recoded **omc** as a series of 3TSs, with no statements appearing more than once per input character [4], [9], and with all 3TSs weighted uniformly (option “-mus”, see Supplement S1, Figure S1, and Example S3). Without application of the “-mus” option, TAXODIUM represents the **omc** as originally proposed by Nelson and Platnick [2].

In the case of ambiguities, an average weight of the 3TSs is assigned to the statement. In cases when all characters of a standard matrix contain ambiguities, weighting is disabled. Before 3TS representations we recommend to treat all gaps and ambiguities of the conventional matrices as a “missing data”.

To demonstrate the capabilities of TAXODIUM, we used the program to generate several types of 3TS matrices, and inferred trees from the resulting matrices with Maximum Parsimony and Maximum Likelihood analyses (Figure 1, Figures S1–S3).

## Discussion

One may note that the exposition of **umc** as a set of 3TSs is somewhat paradoxical: **umc** are not hierarchical and, therefore, are not useful for the 3TS approach. We concluded, however, that **umc**s are useful for the 3TS representations.

As a starting point, we recommend using G- and W-represented, uniformly weighted, and multistate notated 3TS-matrices for Maximum Likelihood estimations (Figure 1, Figure S3) as well as for phenetic algorithms of clustering such as quartet puzzling or Neighbor Joining (NJ). For Parsimony we recommend WS-represented and uniformly weighted 3TS-matrices, especially for cases in which conventional data do not appear clock-like (Figure S2). These options help to prevent grouping by overall similarity (Figure S2, see also Figure S3), as described by Kluge and Farris et al. [12], [13] for some cases of 3TS-matrices produced from non-additive binary data. Also note that the WS 3TS matrices are not useful for the ML analysis.

A few other issues of the 3TS approach [12], [13] do not appear when 3TS matrices are used as an input for an ML analysis perhaps due to the absence of problems posed by long-branch attraction. However, we would like to point out, that a comprehensive implementation of the 3TS analysis in the ML frames will require more work (Mavrodiev et al, in prep.). Future points to address include the 3TS representations of constant and apomorphic characters (e.g., the 3TSs of the kind: (ABC)), as well as applying different substitution models to estimate the likelihoods of the individual 3TSs (Mavrodiev et al, in prep.).

## Supporting Information

Figure S1 Example of MP analysis of 3TS represented omc-matrix. **A.** Single most parsimonious trees (length  = 311, CI  = 0.6045, RI  = 0.3422) recovered from an MP analysis (Wagner parsimony) of conventional male, sex-averaged, and sex-combined allometic cranoidental data matrices for extant African papionins [17], see Figure 10C, Supplemental matrix “mmc4.nex”. Only additive (ordered) multistate characters (## 1–17, 19–44, 46–69, 71–73, 75–136, 138–141 & 143) were included in the matrix, which numbered 137 in total. MP BS values for nodes receiving greater than 50% support are indicated below the branches. See the Figure 1A legend for specific details of the analysis. **B.** The single, most parsimonious tree (length  = 2612, CI  = 0.6535, RI  = 0.4698) resulting from an MP analysis of a 3TS binary matrix generated by TAXODIUM from the conventional matrix of ordered, multistate allometric characters from Figure S1A [17]. The conventional matrix was initially coded as an additive binary matrix, and later represented as suites of 3TSs [1]; all 3TSs were weighted uniformly, **but only unique 3TSs derived from the single input character were saved as characters in the 3TS matrix** (**option“–mus”**), 1707 in total, all informative (command: **taxodium.exe input_file_name.csv –iom –ob –mus –og –nex**). BS values for nodes receiving greater than 50% support are indicated below the branches. See the Figure 1A legend for details of the analysis.
(EPS)

Figure S2 Example of MP and distance analyses of the conventional and 3TS represented non-clocklike umc-matrices. **A.** Tree resulting from a distance analysis (UPGMA) of a non-clocklike *PHYC* matrix [18]; the sequence of *Oryza* was excluded because of potential issues with parology. The selected distance measure was the “mean character difference” [15]. Selection of other distance measures, such as “uncorrected (“p”)” or “ML measure” with various substitution rates and T/T ratios, resulted the same or similar UPGMA topologies. **B.** One of the seven MP trees (length  = 4081, CI  = 0.3619, RI  = 0.3467) recovered by an MP analysis of a *PHYC* conventional matrix [18], with *Oryza* sequence data excluded. The number of characters was 1228, of which 617 were parsimony informative. All characters were treated as “unordered” (Fitch parsimony). JK values for nodes with greater than 50% support are indicated below the branches. See the Figure. 1A legend for analysis details. **C.** The single, most parsimonious tree (length  = 26305, CI  = 0.8142, RI  = 0.07718) recovered by an MP analysis of a 3TS binary matrix generated by TAXODIUM from a conventional *PHYC* matrix [18]; no *Oryza* sequence data was included. Williams-Siebert (WS) representation was performed, and the value of the outgroup fixed as *Amborella*; all 3TSs were weighted uniformly (command: **taxodium.exe input_file_name.csv –idna –ob –og –nex**). The total number of characters (3TSs) was 264201, all of which all were parsimony informative. JK values for nodes receiving greater than 50% support are indicated below the branches. See the Figure 1A legend for details of the analysis.
(EPS)

Figure S3 Example of ML analyses of the conventional and 3TS represented non-clocklike umc-matrices. **A.** The Maximum Likelihood (ML) tree (log likelihood  = −17154.307487) recovered by an analysis of a conventional *PHYC* matrix (Figure S2A–C). ML BS values for nodes receiving greater than 50% support are indicated below the branches. See the Figure 1C legend for details of the analysis. **B.** The ML tree (log likelihood  = −3664791.847208) recovered by an analysis of the IUB-notated 3TS matrix generated by TAXODIUM for the *PHYC* matrix (Figures S2A–C). G-representation was performed; the operational outgroup was not explicitly defined and, therefore, was not included in the analysis (command: **taxodium.exe input_file_name.csv –idna –odna –phy**). All gaps or ambiguities were treated as a missing data. The total number of characters (3TSs) in the 3TS matrix was 956437, all variable. ML BS values for nodes with greater than 50% support are indicated below the branches. See the Figure 1C legend for details of the analysis. **D.** ML tree inferred with PhyML [19], as implemented in a SeaView (ver. 4.0) [20], using the same matrix from Figure S3B. Shimodaira–Hasegawa (SH) –like branch support [20] was calculated instead of ML BS. The SH-like values of 0.9 and greater are indicated below the branches. The TN93 substitution model was assumed as the best choice.
(EPS)

Example S1 3TS representation of binary characters as implemented in TAXODIUM.
(EPS)

Example S2 3TS representations of the unordered (non-additive) multisite character as implemented in TAXODIUM.
(EPS)

Example S3 3TS representation of ordered multistate character using option “unique 3TSs per input-character” [9] (command “-mus”) as implemented in TAXODIUM.
(EPS)

Supplement S1 TAXODIUM: the list of the commands.
(DOCX)
