# Supplementary material for: TAXODIUM Version 1.0: A Simple Way to Generate Uniform and Fractionally Weighted Three-Item Matrices from Various Kinds of Biological Data
Source: PLoS One. 2012 Nov 21;7(11):e48813. doi: 10.1371/journal.pone.0048813 (PMC3504059; doi:10.1371/journal.pone.0048813)
Supplement: Supplement S1 — TAXODIUM: the list of the commands. (DOCX) [file pone.0048813.s007.docx]

**Supplement 1**

**TAXODIUM ver. 1.0** does not require any installation process. Run the program without arguments to view the command reference. The utility of TAXODIUM is designed for building three-item statement (3TS)-matrices from binary and ordered or unordered multistate characters, with options for uniform or fractional weighting of the resulting statements.

**Command line interface**

The first argument must always include the name of the CSV file containing the input matrix. One or several of the following options (in any order) may follow the input file name. Table 1 details available options.

**Table 1. TAXODIUM v1.0: Options**

| **Option** | **Description** |
| --- | --- |
| **Input symbols** | |
| -ib | input: binary (default) |
| -iom | input: ordered multistate |
| -ium | input: unordered multistate |
| -idna | input: DNA/RNA |
| -ip | input: protein |
| **Methods** | |
| -m3 | method: 3TS (default, G-representation = the value of the outgroup exhaustive |
| -mus | - unique statements **per input statement** only (default: off) |
|  |  |
| **Output symbols** | |
| -ob | output: binary (default) |
| -om | output: multistate |
| -odna | output: DNA/RNA |
| -op | output: protein |
| **Fractional weights** (output **NEXUS files only**) | |
| -fw | print fractional weights (default: off) |
|  |  |
|  | **Outgroups** |
| -og | Explicate the operational outgroup (default: off) |
| **Output formats** | |
| -phy | enable PHYLIP output (default: on if no other output selected) |
| -nex | enable NEXUS output (default: off) |
| -csv | enable CSV output (default: off) |
|  |  |

**Table 2 TAXODIUM v1.0: Input file-symbols**

| **Input option** | **Symbols** |
| --- | --- |
| Binary | 0 1 |
| Ordered multistate | 0 1 2 3 4 5 6 7 8 9 : < = > @ A B C D E F G H I J K |
| Unordered multistate | 0 1 2 3 4 5 6 7 8 9 : < = > @ A B C D E F G H I J K |
| DNA/RNA (IUB-codes) | A C G T U R Y S W K M B D H V |
| Protein (IUPA-codes) | A C D E F G H I K L M N P Q R S T V W Y |

**Example 1**

Input matrix format example is shown below:

taxonA,0,0

taxonB,=,0

taxonC,>,3

taxonD,@,4

taxonE,@,6

The first (or leftmost) column contains the names of taxa, and subsequent columns contain characters. Symbols allowed for each input option are shown in Table 2.

**Additionally, the input file can contain a pre-defined outgroup taxon name (W-representation).**  The outgroup must be included as the **last line** of the input file, and in the following format:

Out,taxonB

In the example above, “Out” is a reserved keyword. When preparing an input file with a pre-defined outgroup taxon, “Out” is presented in its own column, and “taxonB” (the name of the outgroup taxon) is presented in a second column in the input CSV file. The word “Out” should not be part of any taxon name.

**Note, that there is no special command for W representation! Simply specify the outgroup taxon on the last line of the input CSV file as previously described.**

**Example 2**

G- representation with binary 3TS matrix output from a standard DNA matrix is output as a simplified NEXUS format with an added outgroup; all 3TS are fractionally weighted:

**taxodium.exe input_file_name.csv -idna -ob -og -fw nex**

**Please note that the command line interface may change in future versions.** Please see the documentation provided with each version of the utility for complete details.

**Limitations and performance**

Currently, the maximum number of taxa in the input matrix must not exceed 5000, and the maximum number of characters must not exceed 100000. These values can be modified in the source code if necessary. The output matrix is constructed entirely using the computer's RAM prior to writing to disk. If a computer has enough RAM to accommodate the entire output matrix, processing will occur with maximum performance. However, if the amount of RAM is insufficient, a typical operating system (such as Windows or Linux) will attempt to use disk swapping and computing performance will be severely compromised. Even cases of insufficient RAM and disk swapping, the program will typically finish processing. However, if the size of the disk swap file is not sufficient, TAXODIUM will report a memory allocation error and display the amount of memory required to accommodate the output matrix. If this scenario should occur, the user will need to increase the size of the disk swap file and rerun the utility.
